# A Comprehensive Review of Biomarker Sensors for a Breathalyzer Platform

**DOI:** 10.3390/s24227263

**Published:** 2024-11-13

**Authors:** Pardis Sadeghi, Rania Alshawabkeh, Amie Rui, Nian Xiang Sun

**Affiliations:** 1 W.M. Keck Laboratory for Integrated Ferroics, Department of Electrical & Computer Engineering, Northeastern University, Boston, MA 02115, USA; sadeghi.p@northeastern.edu (P.S.);; 2 Winchester Technologies LLC, Burlington, MA 01803, USA

**Keywords:** molecularly imprinted polymers, biosensor, non-invasive, nanomaterial, volatile organic compound, biomarker, disease

## Abstract

Detecting volatile organic compounds (VOCs) is increasingly recognized as a pivotal tool in non-invasive disease diagnostics. VOCs are metabolic byproducts, mostly found in human breath, urine, feces, and sweat, whose profiles may shift significantly due to pathological conditions. This paper presents a thorough review of the latest advancements in sensor technologies for VOC detection, with a focus on their healthcare applications. It begins by introducing VOC detection principles, followed by a review of the rapidly evolving technologies in this area. Special emphasis is given to functionalized molecularly imprinted polymer-based biochemical sensors for detecting breath biomarkers, owing to their exceptional selectivity. The discussion examines SWaP-C considerations alongside the respective advantages and disadvantages of VOC sensing technologies. The paper also tackles the principal challenges facing the field and concludes by outlining the current status and proposing directions for future research.

## 1. Introduction

VOCs are gaseous species known to have high vapor pressure under normal room conditions, causing them to evaporate easily [1]. These compounds are abundant in human breath, skin, and bodily fluids, providing valuable chemical, physical, and biological information crucial for non-invasive medical diagnostics [2,3]. Several methods have shown that disease-related VOCs can be directly detected in body fluids [4]. Table 1 summarizes a tentative chemical identification of VOCs associated with various non-infectious diseases, while Table 2 summarizes VOCs related to infectious diseases. Among the physiological sources of VOCs, exhaled breath is considered one of the least complex for analysis due to its simpler gas matrix. The fundamental principle of exhaled breath analysis is that disease-indicative metabolites circulate within the vascular system, and VOCs are consistently released into the breath following alveolar exchange from the blood. Gas exchange occurs in both the airways and the alveoli. Low-solubility VOCs primarily exchange in the alveoli, whereas highly soluble VOCs, especially polar ones, mainly exchange in the airways. Those with moderate solubility affect both regions. The profile of VOCs in the breath is influenced by their blood concentration and the duration that these compounds remain in the lung and airway tissues after inhalation and exhalation [4,5].

VOC sensing faces challenges, primarily because most VOCs are non-reactive and occur at low concentrations, often close to or below the detection limits of conventional analytical devices. Mass spectrometry (MS), a powerful tool in scientific and industrial research, is effective for analyzing, quantifying, and identifying a wide range of compounds, making it suitable for detecting VOCs. Typically paired with liquid or gas chromatography (GC), MS can detect arrays of known and unknown chemicals. The conventional gas chromatography–mass spectrometry (GC-MS) approach, while robust and adaptable, faces several challenges. These include preserving the integrity of samples at the time of collection, where some compounds may degrade, be altered during storage, or not be collected efficiently or selectively. Moreover, both sample preparation and analysis are time-consuming and must be conducted off-line in a laboratory setting, which adds complexity and cost to the process. This is particularly problematic when results are needed in real-time and in a cost-effective manner. To address these limitations, a variety of other sensors have been developed; however, many still fall short of the sensitivity, selectivity, and reliability needed for analyzing complex VOC mixtures in real samples [6,7]. Advances in sensor technology, particularly those incorporating nanomaterials, are pivotal to meeting clinical standards. Nanomaterials offer several benefits due to their high surface-to-volume ratios, which ensure high sensitivity and fast response times. The adjustable chemical and physical properties of nanomaterials allow for increased specificity while combining various nanomaterials can provide synergistic effects. Additionally, the compatibility of nanomaterials with large-scale manufacturing processes facilitates the development of portable, cost-effective devices [8,9,10,11]. The ideal VOC sensor would offer high sensitivity, excellent selectivity, rapid response and recovery times, robust stability and reproducibility, and the ability to operate at room temperature [12,13,14,15].

**Table 1:** Summary of main VOCs for non-infectious diseases.

Disease	VOC Biomarker	Sample Source	Concentration (Normal)	Concentration (Diseased)	Reference
Neurological Diseases					
Alzheimer’s Disease	1,1-oxybis-octane, 1-chloro-nonadecane, 1-methyl-2-(1-methylethyl)-benzene, 1-methylpropyl-cyclooctane, 2,2,4,6,6-pentamethyl-heptane, 2,2-dimethylpropanoic acid, 2,3-dimethyl-heptane, 2,3,5-trimethyl-hexane, 2,4-dimethyl-1-heptene, 2,5,6-trimethyl-octane, 2,6,10-trimethyl-dodecane, 2,6,10,14-tetramethyl-hexadecane, 2,6-octadien-1-ol, 2-butyl-1-octanol, 2-ethylhexyl tetradecyl ester, 3-ethyl-2,2-dimethyl-pentane, 3,7-dimethyl-decane, 3,7-dimethyl-propanoate (E), 4-methyl-octane, 5-ethyl-2-methyl-octane, Butylated HydroxyToluene, Dodecane, Formaldehyde, Oxalic Acid, Propyl-Benzene, Styrene	Breath	0.2–0.8 ppb	1.0–2.5 ppb	[16,17]
Parkinson’s Disease	1-Methyl-3-(1-Methylethyl)-Benzene, 2,3-Dimethyl-Heptane, 2,3,5-Trimethyl-Hexane, 2,3,6,7-Tetramethyl-Octane, 3,7-Dimethyl-Decane, 5-Ethyl-2-Methyl-Octane, Butylated Hydroxytoluene, Decamethyl-Cyclopentasiloxane, Ethylbenzene, Hexadecane, Styrene	Breath	0.2–0.8 ppb	1.0–2.5 ppb	[16,17]
Multiple Sclerosis	5-Methyl-Undecane, Hexanal	Breath	0.022–0.026 ppm	0.009–0.01 ppm	[16,18]
Epilepsy	2-Acetyl-Pyrroline, 2-Acetylpyrrole, 2-Butanone, 2-Heptanone, 2-Pentanone, 2,3,5-Trithiahexane, 3,4-Dehydro-Exo-Brevicomin, Acetophenone, Dimethyl Trisulfide, Disulfide, Menthone, Methanethiol, Methane, Nitro-, Trimethylamine	Feces, Saliva, Urine	Not Reported	Not Reported	[19]
Malignant Diseases					
Lung Cancer	1-(1-Ethoxyethoxy)-pentane, 1,1-Diethoxy-3-methylbutane, 1-Butanol, 1-Ethyl-2-methylbenzene, 1-Hexadecylindane, 1-Propanol, 1,2,4-Trimethylbenzene, 1,4-Pentadiene, 2,2-Dimethyl-hexanal, 2,2,6-Trimethyloctane, 2,3,3-Trimethylpentane, 2,3,5-Trimethylhexane, 2,4-Decadien-1-ol, 2,4-Dimethyl-1-heptene, 2,4-Dimethylheptane, 2,4-Dimethylundecane, 2,5-Dimethyl-furan, 2-Butanol, 2-Butanone, 2-Ethyl-1-hexanol, 2-Ethyldodecanal, 2-Ethyldodecanol, 2-Heptanone, 2-Hydroxyacetaldehyde, 2-Methyl-1-decanol, 2-Methyl-2-butenal, 2-Methylbutanal, 2-Methylbutane, 2-Methylfuran, 2-Methylheptane, 2-Methylpentane, 2-Methylpropanal, 2-Pentanone, 2-Phenyl-2-Butanone, 3,4-Dimethylheptane, 3,7-Dimethyl-decane, 3-Heptanone, 3-Hydroxy-2-butanone, 3-Methyl-3-Hexanol, 3-Methylbutanal, 3-Octanone, 4-Heptanone, 4-Methyloctane, Acetaldehyde, Acetone, Acetic acid, Acetonitrile, Amphetamine, Benzaldehyde, Benzene, Butanal, Butane, Butanoic acid, Butyl acetate, Cyclohexane, Cyclohexanol, Cyclohexanone, Decanal, Dodecane, Dimethyl sulfide (dimethyl sulphide), Dimethyl trisulfide, Dimethylsilanediol, Eicosenamide, Ethanol, Ethyl acetate, Ethylbenzene, Formaldehyde, Heptadecane, Heptanal, Heptane (n-heptane), Hexanal, Hexane, Hydrogen cyanide, Hydrazine, Interleukin-6, Isobutane, Isoprene, Linear and branched hydrocarbons C8–C24, Methyl acetate, Methyl vinyl ketone, Methylcyclopentane, Methacrylaldehyde, Methane, Methanol (methyl alcohol), Methanethiol, Naphthalene, Octanal, o-Xylene, Pentanal, Pentane (n-pentane), Pentamethyl heptane, Pentene, Phenol, p-Cresol, p-Xylene, Propanal, Propane, Propanol, Propene, Propionaldehyde, Propylbenzene (n-propyl benzene), Styrene, Thymol, Thiophene, Tetradecane, Tetrahydrofuran, Toluene (methylbenzene), trans-2-Hexenal, trans-2-Heptenal, trans-2-Nonenal, Trimethylamine, Trimethylbenzene, Undecane, 3,4-dehydro-exo-brevicomin	Breath, Blood, Urine	<35 ppb	Not Reported	[16,17,20,21,22,23,24,25,26,27,28,29]
Pancreatic Cancer	1-(Methylthio)-Propane, 1-Butanol, 1-Decene, 1-Heptene, 1-Nonene, 1-Octene, 2-Butanone, 2-Ethyl-1-Hexanol, 2-Nonene, 2-Pentanone, 2,6-Dimethyl-Octane, Acetaldehyde, Acetone, Acetone Dimer, Acetoin, Amylene Hydrate, Ammonia, Acrylonitrile, Benzaldehyde, Benzene, Butoxymethylbenzene, Carbon Disulfide, Dimethyl Sulfide, Ethane, Ethanol, Formaldehyde, Hydrogen Sulfide, Isoprene, Isopropyl Alcohol, n-Hexane, Nitro-, Nonanal, Pentane, Pentylbenzene, Sulfur Dioxide, Tetradecane, To1uene, Triethylamine, Trimethylamine (TMA)	Bile, Blood, Breath, Urine	1–5 ppb	>5 ppb	[30,31,32,33,34,35,36,37,38]
Gastric Cancer	1,2,3-Trimethylbenzene, 1,3-Dioxolan-2-One, 1,3-Dioxolane-2-Methanol, 1,3-Propanediol, 1-Hexanol, 2-Butoxy-Ethanol, 2-Ethyl-1-Hexanol, 2-Methylhexane, 2-Methylpentane, 2-Pentyl Acetate, 2-Propanol, 2-Propenenitrile, 3-Methyl-2-Pentanone, 3-Methylhexane, 3-Methylpentane, 4-Ethyl Guaiacol, 4,5-Dimethyl-Nonane, 4-Methyl-3-Hexanone, Acetic Acid, Aceticamide, Acetone, Butanal, Butanoic Acid, Decanal, Decene, Dodecane, Dimethyl Disulphide, Ethanol, Ethylene, Furfural, Heptanal, Hexadecane, Hexanal, Hexanol, Isoprene, Menthol, Methyl Phenol, Methylisobutylketone, Naphthalene, Nonanal, Octanal, Pentanal, Pentanoic Acid, Phenol, Phenyl Acetate, Phenylacetic Acid, p-Cresol, Pivalic Acid, Propanal, Styrene, Tetradecane, Thiophene, Tolualdehyde, m-Xylene	Breath	1–116 ppb	>200 ppb	[20,39,40,41,42]
Head and Neck Cancer	1-Butanol, 1-Decen-3-One, 1-Heptene, 1-Methyl-4-2-Methylpropyl-Benzene, 1,1,4,5,6-Pentamethyl-2,3-Dihydro-1H-Indene, 1,3-Bis-(1,1-Dimethylethyl)-Benzene, 2-Butanone, 2-Ethenylfuran, 2-Ethyl-1-Hexanol, 2-Methyl-5-(Methylthio)Furan, 2-Methylbutanal, 2-Methylbutanoic Acid, 2-Methylfuran, 2,2-Dimethyl-Decane, 2,2-Dimethyl-Propanoic Acid, 2,3-Dichloro-Benzeneamine, 2,4-Dimethylfuran, 2,5-Dimethylfuran, 2,6-Dimethyl-7-Octen-2-Ol, 3-Heptanone, 3-Hexanone, 3-Methyl-2-Pentanone, 3-Methylbutanal, 3-Methylfuran, 4-Ethyl-1,3-Benzenediol, 4-Heptanone, 4,6-Dimethyl-Dodecane, 4-Methyl-2-Heptanone, 4-Tolualdehyde, Acetic Acid, Acetone, Benzaldehyde, Benzyl Alcohol, Benzyl Chloride, Camphor, Decamethylcyclopentasiloxane, Dodecane, Dimethyl Disulphide, Dimethyl Trisulfide, Ethylbenzene, Furan, Hydrogen Cyanide, Hexanal, Hexanone, Isoprene, Linalool, Limone, Methyl Ethyl Ketone, Methyl Methacrylate, 2-Methylthiophene, Nonanal, Octanal, p-Cresol, p-Xylene, Phenol, Propanal, Styrene, Tetrahydrolinalool, Terpinolen, Thiophene, Trans-Calamenene, Toluene	Breath, Urine	>1 pbb	<1 pbb	[16,43,44,45]
Breast Cancer	1,1,3,3-Tetramethylurea, 2-Butanone, 2-Butyloctanol, 2-Cyclohexen-1-One, 2-Dodecanone, 2-Ethyl-1-Hexanol, 2-Butoxy-ethanol, 2-Heptanone, 2-Methylbutanoic Acid, 2-Methyl-1,2-Bis(Trimethylsiloxy)-Propane, 2-Methyl-1-Propenylbenzene, 2-Methylfuran, 2-Nonanone, 2-Pentanone, 2-Pentylfuran, 2,2-Dimethylbutane, 2,2-Dimethyl Decane, 2,3-Dimethylhexane, 2,4-Dimethylbenzaldehyde, 2,5-Dimethylfuran, 2,6-Dimethyl-2,4,6-Octatriene, 3,3-Dimethyl Pentane, 3,4-Dimethyl-2,4,6-Octatriene, 3,4-Dimethyl-2-Hexanone, 3-Hexanone, 3-Methyl-2-Heptanone, 3-Methyl-3-Buten-1-Ol, 3-Methyl-3-Butenol, 3-Methylpyridine, 3-Methoxy-1,2-Propanediol, 4-Ethyl-1,3-Benzenediol, 4-Hydroxybutanoic Acid, 4-Methyl-2-Heptanone, 5-Butylnonane, 5-Methyl-3-Hexanol, Alkanes, Allyl Isothiocyanate, Ammonium Thiocyanate, Acetic Acid, Acetaldehyde, Acetone, Acetophenone, Benzaldehyde, Benzenecarboxylic Acid, Benzoic Acid, Benzophenone, Benzocyclobutene, Benzyl Alcohol, Butyl Acetate, Cadalene, Carbon Disulphide, Cyclohexanol, Cyclohexanecarboxylic Acid, Cyclohexanone, Cyclooctylmethanol, Cyclopentane, Cyclopentanone, D-Limonene, Decanoic Acid, Dodecane, Dimethyl Disulfide, Dimethyl Trisulfide, Dimethylacetamide, Ethanol, Ethyl Acetate, Ethyl Benzene, Ethyl Ether, Ethyl Propanoate, Ethylene Carbonate, Furan, Furfural, Guaiacol, Heptanoic Acid, Hexadecane, Hexanal, Hexanoic Acid, Hexamethyldisilane, Isobutyric Acid, Isoprene, Isoterpinolene, L-Alanine, L-Isoleucine, L-Proline, Limonene, Menthomenthol, Menthol, Methanol, Methanethiol, Methyl Acetate, Methyl Chloride, Methyl Salicylate, Methylacrylic Acid, Naphthalene, Nonanal, Octanoic Acid, Pentane, Pentanoic Acid, Phellandranal, Phenol, Polycyclic Aromatic Hydrocarbons, Prehnitene, Propanal, Propanoic Acid, Pyrrolidine, Styrene, Tetradecane, Tetrahydrolinalool, Tetramethylsilicane, Toluene, Trans-2-Butene Oxide, Trimethyl Trisulfide, 1,2-Propanediol, 1,2,4-Trimethylbenzene, 1,3-Dimethylbenzene, 1,4-Cineole, 1,4-Dimethoxy-2,3-Butanediol, 1,5-Cyclohexadiene, 1-Hexadecanol, 1-Octanol, 1-Propanol, 2,2-Dimethylbutane, 2,3,4-Trimethylheptane, 2,3,6-Trimethyloctane, 2-Ethenylfuran, 2-Methoxythiophene, 3-Ethylcyclopentanone, 3-Methyl-2-Pentanone, 3-Methylfuran, 3-Methylthiophene, 4,6-Dimethyl-Dodecane, 4-Methyl-2-Hexanone, 6-Methyl-5-Hepten-2-One, α-Pinene, α-Terpinolene.	Breath, Cell Lines, Urine	4–10 ppm	8–50 ppm	[16,20,29,39,46,47,48]
Colon Cancer	1,1,4,4-Tetramethyl-2,5-Dimetylene-Cyclohexane, 1,2-Dihydro-1,1,6-Trimethyl-Naphthalene, 1,2-Pentadiene, 1-Octanol, 1,3-Dimethylbenzene, 1,4-Dimethylbenzene, 1,3-Bis(1-Methylethenyl) Benzene, 2-Amido-5-Isopropyl-8-Methyl-1-Azulenecarbonitrile, 2-Ethylhexanol, 2-Methyl-3-Phenyl-2-Propenal, 2,2-Dimethyldecane, 2,7-Dimethylquinoline, 3-Ethylpentane, 3-Hydroxy-2,4,4-Trimethylpentyl 2-Methylpropanoate, 3-Methylpentane, 4-Ethyl-1-Octyn-3-Ol, 4-Methyl-2-Pentanone, 4-Methylphenol, 4-Methyloctane, 6,10-Dimethyl-5,9-Undecadien-2-One, 6-t-Butyl-2,2,9,9-Tetramethyl-3,5-Decadien-7-Yne, 6-t-Butyl-2,2,9,9-Tetramethyl-3,5-Decadien-7-Yne, Acetic Acid, Acetaldehyde, Acetone, Acetyloxime-Pyridine Carboxaldehyde, Allylisothiocyanate, Ammonia, Anisole, Benzoic Acid, Benzaldehyde, Butanol, Butanoic Acid, Butyl Hydroxy Toluene, Butylated Hydroxytoluene, Carbon Disulfide, Cyclohexane, Cyclohexanone, Cyclooctylmethanol, Dodecane, Dodecanoic Acid, Ethanol, Ethyl Acetate, Ethylbenzene, Ethylhexanol, Ethylaniline, Hexana, Heptanal, Hydrogen Sulphide, Indole, Methylbenzene, Methylcyclopentane, Methylcyclohexane, Nonanal, Octanoic Acid, Pentanoic Acid, Phenol, p-Cymene, Propanal, Propanol, Tetradecane, Tridecane.	Breath, Blood, Feces, Urine	Not Reported	Not Reported	[16,20,48,49]
Prostate Cancer	1-(2,4-Dimethylphenyl)-3-(Tetrahydrofuryl-2)Propane, 2-Acetylpyridine, 2-Butanone, 2-Eethylhexanol, 2-Hexanone, 2-Pentanone, 2,2-Dimethyl Decane, 2,5-Dimethylbenzaldehyde, 3-Carene, 3,5-Dimethylbenzaldehyde, 3-Methylphenol (m-Cresol), Acetaldehyde, Aldehydes, Estradiol, Furan, Hexanal, Indole, Isoterpinolene, Linalool, Methyl Butyrate, Phenol, Phenylacetaldehyde, Phenylpropionaldehyde, Pentanal, Propyl Propionate, Theaspirane, Terpinen-4-ol, Toluene, p-Xylene, 2,6-Dimethyl-7-Octen-2-Ol, 2-Amino-5-Isopropyl-8-Methyl-1-Azulenecarbonitrile.	Breath, Urine	Not Reported	Not Reported	[16,20,50,51,52]
Liver Cancer	1-Octen-3-Ol, 1,4-Pentadiene, Acetic Acid, Acetone, Allyl Methyl Sulfide, Camphene, Cyclopentane, Dimethyl Sulfide, Ethanol, Hexanal, Methane-Sulfonyl Chloride, Methylene Chloride, Octane, Phenol, 2-Pentanone, 2,3-Di-Hydro-Benzofuran	Breath, Blood	0.5–1.5 ppb	1.0–4.5 ppb	[16,17,53]
Gastro-Esophageal Cancer	Acetaldehyde, Acetone, Acetic Acid, Ethanol, Ethyl Phenol, Formaldehyde, Hexanoic Acid, Hydrogen Cyanide, Hydrogen Sulfide, Methanol, Methyl Phenol, Phenol, Propanol.	Breath, Urine	1–28 ppbv	>28 ppbv	[25,42,54]
Leukemia	2,4-Dimethylheptane, Benzene, 4-Methyl decane, Chloroform, 3,7-Dimethyl dodecane, Hexanol, Cyclohexanol, Hexadecane, p-Cresol, Dimethyl Disulphide	Cell Lines, Urine	Low Levels	2× to 3× higher	[50,55,56]
Renal Cell Carcinoma	2-Oxopropanal, (1Z)-1-Propen-1-ylbenzene (a-methylstyrene), 2,5,8-Trimethyl-1,2,3,4-tetrahydronaphthalen-1-ol, [(3S,8R,9S,10R,13S,14S)-10,13-Dimethyl-17-oxo-1,2,3,4,7,8,9,11, 12,14,15,16-dodecahydrocyclopenta[a]phenanthren-3-yl] hydrogen sulphate (DHEA-S), 2-Oxopropanal (Pyruvaldehyde), 2-Methylpropan-2-ol, 2-Ethoxy-2-methylpropane, 2-Methylpropan-1-ol (Isobutanol), 2-Methylbutan-2-ol, Pentane-2-one, 2,2,5,5-Tetramethyltetrahydrofuran, 1-Methyl-1,4-cyclohexadiene, 4-Methylheptan-2-one, Phenol, 2-Pentylfuran†, 3,7,7-Trimethylcyclohept-3-ene (2-Carene), 2,2-Dimethylpropionic acid butyl ester, 6-Methyl-5-hepten-2-ol, 1-Methyl-4-(1-methylethenyl)-cyclohexene (Limonene), 1,2,3,4-Tetrahydro-1,5,7-trimethylnaphthalene†, 1-(2-Methylphenyl)-2-propen-1-one, 1,1,6-Trimethyl-1,2-dihydronaphthalene (TDN), 2-Methoxy-4-prop-2-enylphenol (Eugenol), (E)-1-(2,3,6-Trimethylphenyl)buta-1,3-diene, 1,1,5,6-Tetramethyl-1,2-dihydronaphthalene, 2,5,8-Trimethyl-1,2,3,4-tetrahydronaphthalen-1-ol, [(2E,4E,6E,8E)-3,7-dimethyl9-(2,6,6-trimethylcyclohexen-1-yl)nona-2,4,6,8-tetraenyl] acetate (Retinol acetate), [(3S,8R,9S,10R,13S,14S)-10,13-Dimethyl-17-oxo1,2,3,4,7,8,9,11,12,14,15,16-dodecahydrocyclopenta[a] phenanthren-3-yl] hydrogen sulphate (DHEA-S)	Urine	Not Reported	Not Reported	[57]
Metabolic Diseases					
Diabetes	Acetone, Isoprene, Isopropanol, Ethanol, Methyl nitrate	Breath	0.85–1.8 ppm	0.39–0.8 ppm	[16,17,20,25]
Hyperglycemia	Ethylbenzene, Methyl Nitrate, Xylene	Breath	Not Reported	Not Reported	[16]
Phenylketonuria	Phenylacetic acid	Sweat, Urine, Skin	Not Reported	Not Reported	[16]
Methionine Malabsorption Syndrome	α-hydroxybutyric acid	Breath, Skin	Not Reported	Not Reported	[16]
Hypermethioninemia	Dimethyl Sulfide	Breath, Urine	Not Reported	Not Reported	[16]
Trimethylaminuria	Trimethylamine	Breath, Sweat	Not Reported	Not Reported	[16]
Other Diseases					
Asthma	2-Hexanone, 3,6-Dimethyldecane, 8-Isoprostane, Ammonia, Decane, Ethane, H2O2, Hexane, Nonal, Nonane, Pentane, Propanol, Tetradecane	Breath	8–16 ppm	10–30 ppm	[16,20,25,46]
Chronic Obstructive Pulmonary Disease	2-Acetylpyridine, 2-Butyloctanol, 2-Dimethyl-heptane, 2-Ethylmethyldecane, 2-Methylbutanoic Acid, 2-Pentanone, 2,4,4-Trimethylpentene, 2,4-Dimethylheptane, 2,6-Dimethyloctane, 3,4-Ethylmethylhexane, 3-Hexanone, 3-Methylcyclopentanone, 4-Heptanone, 4-Methyl-Octane, 4,7-Dimethyl-undecane, Acetaldehyde, Alkanes, Aldehydes, An Unidentified C16-Hydrocarbon, Butanal, Butane, Butylatedhydroxytoluene, Cyclohexane, Cyclohexanone, CO, Decane, Dimethyl Disulphide, Δ-Dodecalactone, Ethane, H2O2, Heptanal, Heptane, Hexadecane, Hexylethylphosphonofluoridate, Indole, Isoprene, Isopropanol, Limonene, Methylisobutyrate, Methylpropylsulphide, Nonanal, Nonanoic Acid, Nitro Tyrosine, Octane, Pentane, Propanal, Propanoic Acid, Tetradecane, Vinylpyrazine	Breath	Low or negligible	Elevated levels	[16,20,46,58]
Halitosis	Allyl Isothiocyanate, Allyl Mercaptan, Allyl Thiocyanate, Ammonia, Carbon Disulfide, Dimethyl Amine, Dimethyl Pentasulfide, Dimethyl Sulfide, Ethyl Propyl Sulfide, Hydrogen Sulfide, Methyl Mercaptan, Methyl Thiolacetate, N-Butyric Acid, Propyl Mercaptan, Skatole, Trimethyl Amine, S-Methyl Pentanethioate	Breath, Saliva	250 ppb	400 ppb	[16,59]
Inflammatory Bowel Disease	Acetic Acid, Acetaldehyde, Acetone, Acetonitrile, Acrylonitrile, Ammonia, Butanal, Butane, Butanol, Butyric Acid, Carbon Disulfide, Cyclopentane, Cumene, Decanal, Ethyl Cyanoformate, Ethyl Phenol, Ethane, Ethanol, Hexanoic Acid, Hexanal, Hexadecanal, Isoprene, Methyl Cyclopentene, Methyl Ethyl Ketone, Methyl Nitrate, Methyl Phenol, Methyl Sulfide, Nonanal, Octanal, Octane, Pentadecene, Pentanal, Pentane, Pentanoic Acid, Pentanol, Phenol, Propanoic Acid, Propane, Propanol, Propene, Propyl Ester, Pyridine, 1-Butoxy-2-Propanol, 1-Decene, 1-Heptene, 1-Nonene, 1-Octene, 2,2,4-Trimethylhexane, 2,2,4-Trimethylpentane, 2,4-Dimethylpentane, 3-Methyl-1-Butanol, 3-Methyl-1-Butyl Ester, 3-Methylhexane, Dimethyl Disulfide, Dimethyl Sulfide, Dimethylpyridine, Ethylene, Heptadecane, Hydrogen Cyanide, Hydrogen Sulfide, Limonene, Methanimine, 1-Hydroxy-2-Propanone, Methanol, Nitrous Acid, Toluene, Triethyl Amine, Trimethyl Amine, Trimethylpentane, Undecanal	Breath	Not Reported	Not Reported	[16,48]
Heart failure	NO	Breath	Not Reported	Not Reported	[16]
Hepatic encephalopathy	3-methylbutanol; Limonene; Methyl Mercaptan	Blood, Breath	Not Reported	Not Reported	[16]
Liver failure	Acetic Acid, Acetaldehyde, Ammonia, Ethane, Ethanol, Limonene, Methanol, Methylmercaptan, Pentane, Propionic Acid, Trimethylamine, Dimethyl Sulfide, Carbonyl Sulfide	Breath	2–836 ppb	>1000 ppb	[16,41,60]
Chronic Renal Failure/Uremia	1,8-Cineol, 2-Butanone, 2-Ethyl-1-Hexanol, 2-Methyl Pentane, 2-Methylpropyl Methyl Ketone, 2,4-Dimethyl-Heptane, 2,2,6-Trimethyl-Octane, 3-Carene, 3-Heptanone, Acetaldehyde, Acetone, Acetic Acid Ethyl Ester, Acetophenone, Azulene, Cyclohexanone, Decane, Dihydro-2(3H)-Furanone, Dimethylamine, Dimethyl Selenide, Ethyl Cyclohexane, Ethylene Oxide, Heptane, Isoprene, m-Xylene, Myrcene, n-Nonane, Octadecane, Octanal, o-Xylene, Pentadecane, Phenol Alcohol, p-Xylene, Sulfur Dioxide, Trichloroethene, Trimethylamine, γ-Terpinene	Breath	250 ppb	400 ppb	[16,17,61]
Schizophrenia	1-Hexanol, Carbon Disulfide, Ethane, N-Butylamine, Pentane	Breath	5–140 ppb	Decreased Levels	[16,46,62]
Cystic fibrosis	Hydrogen Cyanide, Methyl Thiocyanate	Breath	0–12 ppb	Not Reported	[25,46,63]

**Table 2:** Summary of main VOC for infectious diseases.

**Disease**	**Biomarker**	**Concentration** **(Normal)**	**Concentration** **(Diseased)**	**Sample** **Source**	**Reference**
COVID-19	2-Butanone, 2,2-Dimethyloctane, 2,2,4-Trimethylheptane, 2-Pentyl Furan, 3,6-Methylundecane, Acetaldehyde, Acetone, Benzaldehyde, Butane, Butene, Butyraldehyde, Camphene, Carbon Dioxide, Decane, Decene, Dimethyldecane, Dodecane, Ethan-1-ol, Ethanol, Ethyl Butanoate, Heptanal, Iodobenzene, Isoprene, Isopropanol, Methylcyclopentane, Methyldecane, Methylpent-2-Enal, Nonanal, Nitrogen Monoxide, Nitrogen Oxide, Octanal, Propanol, Propionic Acid, SARS-CoV-2 RBD Spike Protein, Tridecane, and Trimethyloctane	0.45 to 2.34 ppbv	3 to 10 ppbv	Breath	[64,65,66,67,68,69,70,71]
Influenza	1-Butyl-2-Ethylcyclopentane, 1-Docosene, 1-Decanal, 1-Hexadecene, 1-Octadecene, 1-Tetradecene, 1-Phenoxypropan-2-Ol, 1,2,6,6-Tetramethyl-1,3-Cyclohexadiene, 2-Butanamine, 2-Deoxyecdysone 22-Phosphate, 3-Methyl-Butane, 3-Methyl-Pentane, 4-Quinolinecarboxaldehyde, 4-Tetradecene, 5-(1-Methylpropyl)-Nonane, 6-Dodecene, 6-Methyl-4E-Decene, 7-Hexadecene, 7-Octadecene, 9-Eicosene, 9-Hexadecenoic Acid, Acetaldehyde, Acetone, Alpha-Pinene, Benzoic Acid, Benzoic Acid Alkane Ester I, Benzoic Acid Alkane Ester II, Decanal, Dimethyl-2-Propylcyclohexane, Dodecanal, Dodecyl Nonyl Ether, Ethyl 4-Ethoxybenzoate, Hexanal, Heptane, Homotyrosine, L-Tyrosine Methyl Ester, Linalyl Propionate, Lubiminol, Octadecanoic Acid, Octanal, Propanol, N-Propyl Acetate, Tetradecanal, Tridecadienoic Acid, Tridecynoic Acid, Trimethyl Octene	1–100 ppb	>500 ppb	Breath, Cell Culture	[72,73,74,75]
Tuberculosis	1-Methyl-Naphthalene, 1,2,3-Trimethylbenzene, 1,4-Dimethyl-Cyclohexane, 1-Nitroadamantane, 1-Propynylbenzene, 2-Ethylhexyl Isobutyl Sulfite, 2-Pinene, 2,2,3-Trimethylhexane, 2,2,4,6,6-Pentamethylheptane, 2,3,6-Trimethylheptane, 2,3,6-Trimethylnapthalene, 2,5-Dimethyldecane, 3-Heptanone, 3-Pentanol, 3-Hydroxy-3-Methylbutanoic Acid, 4-Ethyl-2,2,6,6-Tetramethylheptane, 4-Methyl-1-Decene, 4-Tert-Amyl Phenol, Azulene, Benzophenone, Butyl Acetate, Cyclohexane, Cymol, Ethyl Butyrate, Heptanal, Indane, Isopropyl Acetate, Naphthalene, O-Xylene, beta-Phellandrene	Low or negligible	2 to 5 ppbv	Breath, Cell Culture, Urine	[76,77]
Chronic hepatitis	1-Decene, 1-Heptene, 1-Nonene, 1-Octene, 2-Propanol, Acetaldehyde, Acetone, Acrylonitrile, Ammonia, Benzene, Carbon Disulfide, Dimethyl Sulfide, Ethane, Ethanol, Hydrogen Sulfide, Isoprene, Pentane, Triethyl Amine, Trimethyl Amine, 3-Methylhexane	0.45 to 1.2 ppbv	1.08 to 2.08 ppbv	Breath	[75,78,79]
Sinusitis, pneumonia	1-Vinylaziridine, 2-Aminoacetophenone, 2-Methylbutyric Acid, 2-Nonanone, 2-Propanol, Acacetamide, Acetaldehyde, Acetone, Acetoin, Acetic Acid, Benzoic Acid, Benzyl Alcohol, Benzophenone, Butan-1-ol, Butyrolactone, Caprolactam, Dimethylsulfide, Dimethylsulfone, Furfuryl Alcohol, Hydroxyacetone, Indole, Isobutyric Acid, Isovaleric Acid, Methyl Thiocyanate, Nitrogen Oxide, p-Cresol, p-Ethylphenol, Phenol, Propanoic Acid, 3-Hydroxy-2-Butanone, 3-Methyl-1-Butanol, 3-Methylpyrrole, Pyrrole, 1-Undecene	0.5 to 1.5 ppbv	1 to 10 ppbv	Sinus Mucus	[80,81]
Bronchitis, pneumonia	Butraldehyde, octyle acetate, tridecanol, dodecanal, butanoic acid, N-acetyl-S-(4-hydroxy-2-butenyl)-l-cysteine, N-acetyl-S-(2-carbamoylethyl)-l-cysteine, N-acetyl-S-(2-cyanoethyl)-l-cysteine, N-acetyl-S-(N-methylcarbamoyl)-l-cysteine, N-acetyl-S-(benzyl)-l-cysteine, N-acetyl-S-(3-hydroxypropyl-1-methyl)-l-cysteine, Benzene, 1,4-Dichlorobenzene, Ethylbenzene, o-Xylene, Styrene, Trichloroethene, Toluene, p-Xylene	0.5 to 1 ppbv	1 to 2 ppbv	Blood, Urine	[82,83]

The ultimate goal of research in this field is to develop non-invasive, cost-effective diagnostic methods that can replace traditional invasive, complex, and time-consuming approaches. These new methods aim to enable early detection of both infectious and non-infectious diseases, as well as continuous health monitoring, especially in resource-limited areas where traditional methods are not feasible. Beyond healthcare, VOC detection technologies have potential applications in environmental and industrial monitoring, where they could be used to detect pollutants or hazardous substances.

**Scheme 1:**
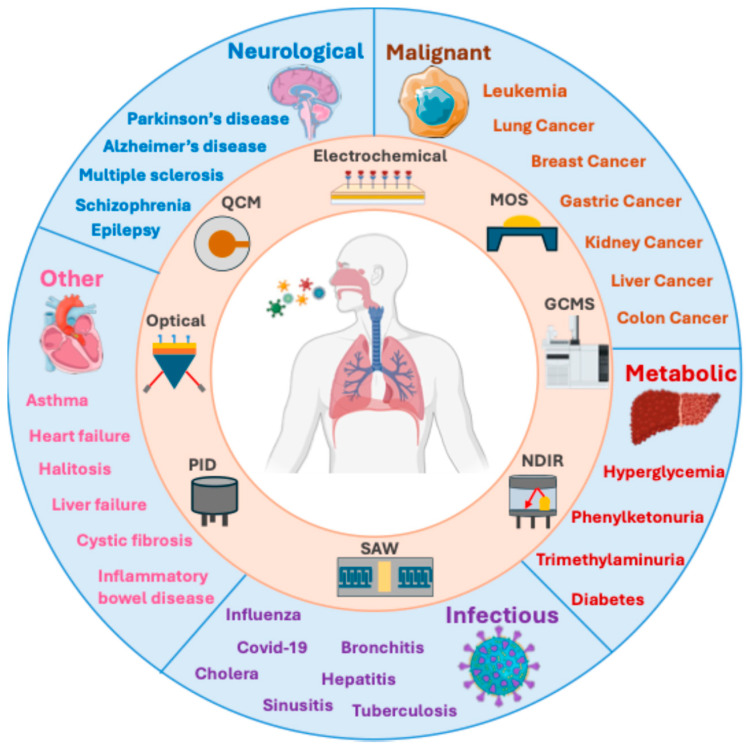
Illustration of VOC-based sensors for disease diagnosis.

This review focuses on innovative analytical methods that go beyond traditional off-line sample collection and laboratory analysis, with a particular emphasis on on-site and in situ techniques, as shown in Scheme 1. It provides an overview of these methods, highlighting significant advancements and their primary functions. Although there are various ways to categorize VOC characterization, this review is organized around the different analytical approaches used for detection. To aid ongoing research, in this review we present a comprehensive summary of biomarkers associated with a range of diseases, based on studies from the past decade involving various biofluids. We also include tables outlining the tentative chemical profiles of VOCs linked to both infectious and non-infectious diseases, identified through spectrometry techniques. These tables offer valuable reference materials for researchers working to bridge the gap between laboratory-based discoveries and practical diagnostic applications.

## 2. Screening Methods for VOC Analysis

A variety of gas sensors and sensor arrays are employed for detecting volatile organic compounds (VOCs). When assessing the efficacy of VOC gas sensors and their respective sensing methodologies, it is vital to consider several indicators that determine their performance. Key metrics include sensitivity—the sensor’s ability to detect the lowest concentration of gases; selectivity—the sensor’s ability to identify specific gases within a gas mixture; reversibility—whether the sensor can return to its baseline state after detecting gases; as well as response time, energy consumption, and fabrication costs. These metrics not only define the sensor’s functionality but also influence its design and application, ensuring that the sensors are not only effective but also economical and durable. The discussion and comparison of these factors will be elaborated in Section 4. Furthermore, for a gas sensor to be suitable for commercial use, it must demonstrate consistent performance over time. Factors contributing to a sensor’s instability include design imperfections, phase shifts in materials, and external environmental impacts. To mitigate these issues, strategies involve selecting materials that are both chemically and thermally stable and implementing advanced technological processes during the sensor’s surface pre-treatment.

The upcoming discussion will provide an in-depth analysis of the sensitivity and selectivity of various VOC sensing methods, underscoring their crucial roles in the precise detection and characterization of volatile organic compounds.

### 2.1. Methods Based on Variation of Electrical Properties

#### 2.1.1. Electrochemical Sensors

Electrochemical sensors detect volatile organic compounds (VOCs) by reducing or oxidizing them through redox reactions at the working electrode interface within an electrochemical cell, which may contain a solid, liquid, or gaseous electrolyte or ionic conductor. The operation of these sensors hinges on the electrochemical reactions at the electrodes, where the transfer of charge and subsequent flow of current directly correlate with the concentration of VOCs. These sensors are versatile in their operational modes, offering amperometry-based detection, which measures the flow of redox current, and potentiometry-based detection, which assesses potential differences. Additionally, they can perform cyclic voltammetry, a technique that provides unique oxidation or reduction peaks for each VOC, enabling highly selective and precise detection. Innovations in electrochemical sensor (ECS) materials have significantly enhanced sensor performance and broadened their applications. ECS materials are specially designed to detect and measure specific chemical substances by facilitating electrochemical reactions that produce measurable electrical signals. For instance, Silverster noted the advantages of using ionic liquids as electrolytes, highlighting their wide potential range and ability to extend sensor lifespan under dry conditions and aiding in device miniaturization [84]. Miha et al. explored the use of polypyrroles as a sensor material, discussing the impact of doping, surface modifications, and side-chain selection on sensor efficiency [85]. Kumar et al. discussed the use of metal–organic frameworks in ECSs, detailing strategies like doping and functionalization to optimize detection capabilities [86]. These sensors are celebrated for their selectivity, accuracy, and reliability in detecting VOCs in various environments, from industrial settings to healthcare. Their ability to perform functions like amperometry, potentiometry, and cyclic voltammetry. Moreover, their versatility extends to applications in air quality monitoring, food safety, and even wearable health devices that monitor biomarkers in bodily fluids like sweat, showcasing their broad utility and the continued advancement in electrochemical sensing technology [87]. The endless selection of sensor geometry, configurations, and electrode materials renders these sensors applicable to many environments while maintaining broadband detection. These sensors have numerous advantages: low detection limit, low power consumption, rapid response times, simple fabrication, and low cost [88]. However, their main limitations are their limited shelf life and low baseline stability, leading to degraded performance over time [89].

Ardisana et al. [90] developed a lactate biosensor using a screen-printed carbon electrode modified with lactate oxidase and a nanocomposite of carbon nanofibers functionalized with platinum. This biosensor exhibited a linear detection range of 25–1500 μM and was successfully applied to lactate measurement in sweat samples. Despite its strong performance, the authors concluded that sweat lactate is not a reliable indicator for determining the lactate threshold.

In a more recent study, Kim et al. [91] introduced a non-invasive mouthguard biosensor, modified with Prussian blue and lactate oxidase, for continuous monitoring of salivary lactate. While this mouthguard biosensor demonstrated significant potential as a wearable device for real-time fitness monitoring, the authors did not establish a correlation between salivary and blood lactate concentrations before or after physical activity.

**Figure 1:**
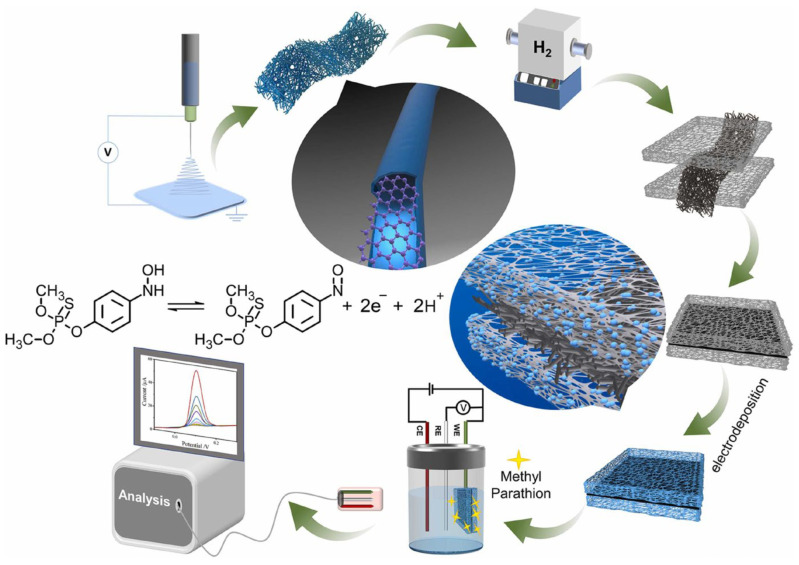
Fabrication of PEDOT/YSZ@rGO/SS electrode [92]. Reprinted from [92], Copyright (2022), with permission from Elsevier.

Lv et al. designed an electrochemical sensor based on zirconia nanofibers for methyl parathion detection which showed lower levels of detection, acceptable recovery, and good stability. Their fabrication process can be seen in Figure 1. While the results are promising, the stability of these sensors needs to be further improved to achieve long-term usability [92].

In summary, electrochemical sensors offer key advantages such as high sensitivity, low power consumption, room temperature operation, and rapid, real-time detection, making them ideal for portable monitoring. However, they have limitations in sensitivity and selectivity, with limited baseline stability and cross-sensitivity to other gases. Additionally, a shorter life and higher maintenance requirements can impact long-term usability. Despite these drawbacks, they remain effective for fast, cost-efficient monitoring applications where very high selectivity is not critical.

#### 2.1.2. Chemiresistors

Chemiresistors, which first gained attention in the 1960s for their potential in VOC sensing, utilize thin films of electrically conductive polymers that are sensitive to the presence of volatile organic compounds (VOCs) in the vapor phase. These films undergo volumetric expansion in response to VOC exposure, a process quantitatively measured through variations in electrical resistance. This swelling effect is reversible, enabling the device to return to its baseline state once the VOCs are no longer present, thus allowing for repeated use without the need to replace any components [93]. Arrays of these miniature, low-energy devices have demonstrated efficacy in detecting multiple chemical contaminants. Notably, chemiresistors are devoid of moving parts and require only basic direct current (DC) circuitry to monitor changes in resistance. These devices are distinguished by several advantages that have facilitated their adoption in commercial settings, such as their straightforward manufacturing process, a diverse array of effective sensing materials, and simple operational mechanisms.

**Figure 2:**
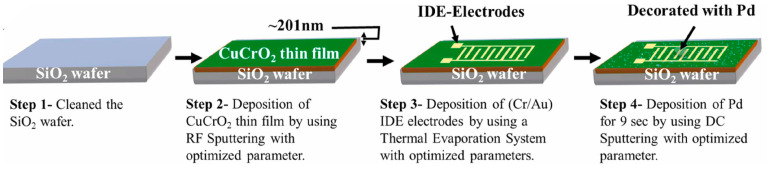
The fabrication process of the device is illustrated in the schematic diagram [94]. Reprinted from [94], Copyright (2022), with permission from Elsevier.

Over the years, the materials used in chemiresistors for detecting VOCs have broadened significantly. These include metal oxides, metal nanoparticles, conductive polymers, inorganic semiconductors (such as phosphorene), carbon-based materials, hybrids and composites, and ionic conductors. For instance, chemiresistors that incorporate monolayer-capped nanoparticles modify their resistance by altering the spacing between nanoparticles as they interact with VOCs, thereby providing a measure of the VOC concentration or composition. Conversely, resistance changes in chemiresistors using conductive polymers are driven by processes like swelling, doping, and protonation when exposed to VOCs. Kumar et al. showed that using the metal oxide palladium increased the surface-to-volume ratio and improved sensing performance [94]. Figure 2 showcases the fabrication steps of the device.

Nanomaterials remain a popular choice in this research area due to their large surface areas and enhanced sensitivity, highlighting the continuous evolution and application of chemiresistors in sensing technologies [95,96,97]. Srivastava et al. produced successful results incorporating graphite oxide to fabricate toluene sensors, which showed excellent selectivity and sensitivity while maintaining fast response and recovery times [98]. While the use of nanomaterials enhances the sensors’ sensitivity, it also introduces challenges such as non-uniform surfaces, complex processes, and high costs. Materials like graphene are also susceptible to oxygen absorption, blocking activation sites and degrading performance. Methods to counter these effects, such as UV irradiation, have been shown to be promising in improving the lifespan and performance of these altered sensors [99]. Nonetheless, chemiresistors have shown promise in various fields.

Metal oxide semiconductor (MOS) sensors are a specific type of chemiresistor and one of the most prevalent devices for detecting volatile organic compounds (VOCs), often used in electronic noses. These sensors utilize a metal oxide thin film whose resistance and conductivity change upon interaction with ambient gases. They measure oxidizing or reducing compounds based on the chemisorption of oxygen on the semiconductor surface, which alters the electron availability. N-type MOS (e.g., TiO_2_) decreases in conductivity when exposed to oxidizing VOCs and increases with reducing ones, while P-type sensors (like NiO) operate in the reverse manner. The performance of MOS sensors can be influenced by composition, surface area, doping, temperature, and humidity. Their utility extends across various applications, including detecting bacterial infections and viral diseases in plants [100,101,102].

In a broader context, research on MOS sensors has significantly advanced, focusing on enhancing their selectivity and sensitivity through innovative approaches such as doping with rare earth elements or using nanostructured metal oxides. These advancements have led to the development of sensors capable of operating at lower temperatures and providing more precise detections. Despite their broad application and improvements in sensor technology, MOS sensors still face challenges like sensor drift due to environmental changes and a generally slow response time due to the kinetics of gas adsorption and desorption. Recent research has focused on enhancing MOS sensors through innovative fabrication methods, including nanostructuring which improves selectivity and operational temperature. Acharyya et al. discriminated VOCs using a tin-oxide-based hollow sphere MOS, calculating kinetic interactions for enhanced sensitivity [103]. Fois et al. increased selectivity by doping metal oxides with rare earth elements [104], while Pargoletti and Cappelletti achieved performance gains by integrating carbonaceous materials [105]. Additionally, Baur et al. explored temperature-cycled operations and machine learning to reveal MOS’s hidden potential [106], and Gao et al. improved the MOS structure with hierarchically porous designs [107]. Despite their widespread use, MOS sensors have limitations, such as high power consumption due to their need for high operational temperatures, slow adsorption and desorption rates leading to lengthy measurement times, and susceptibility to environmental factors like temperature and humidity.

**Figure 3:**
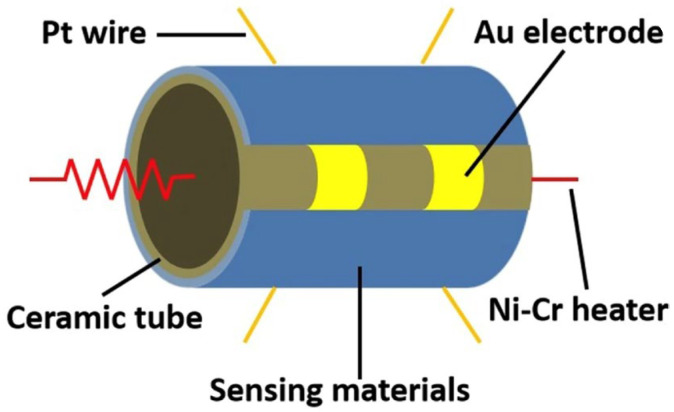
Schematic structure of the tin oxide gas sensor [108]. Reprinted from [108], Copyright (2019), with permission from Springer Nature Open Access Collection.

Certain nanostructures decrease the melting temperature of MOS, potentially leading to deformation. Damage is also more likely to occur during fabrication due to the nanostructure’s high surface-to-volume ratio. Song et al. explored using tin oxide nanoflowers to improve sensing properties while maintaining a simple and low-cost preparation. The schematic structure of the gas sensor device is presented in Figure 3 [108].

Nonetheless, they remain valuable in detecting a broad range of VOCs and some inorganic gases like CO and NOx, although their long-term stability can be an issue [109]. They also offer a balance of durability, portability, and cost-effectiveness, with simple operation and real-time response capabilities that make them ideal for non-destructive, on-site applications. While they can detect a broad spectrum of gases, their relatively low sensitivity and selectivity, along with their sensitivity to humidity and temperature fluctuations, can limit performance in complex environments. Moreover, their need for high operating temperatures and fragility during preparation reduces their flexibility. Nevertheless, chemiresistors remain a practical choice for gas detection where simplicity, portability, and cost-efficiency are prioritized.

### 2.2. Methods Based on Variations of Other Properties

#### 2.2.1. Quartz Crystal Microbalance (QCM)

**Figure 4:**
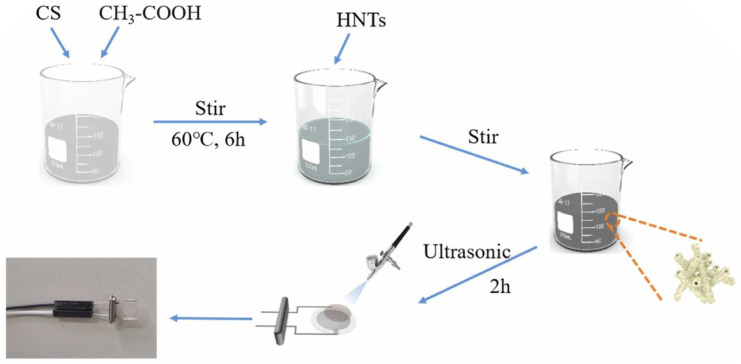
Preparation process of the CS-HNTs film-coated QCM humidity sensor [112]. Reprinted from [112], Copyright (2023), with permission from Elsevier.

Quartz Crystal Microbalance (QCM) technology is widely recognized for its affordability and accessibility. Operating on the principle of resonance frequency, QCM devices excel in both liquid and gas sensing applications, offering versatility across industries, including environmental monitoring, food safety, and healthcare diagnostics. A voltage is applied to a quartz crystal, initiating oscillation via the reverse piezoelectric effect. The crystal is subsequently coated with a film analogous to those utilized in Surface Acoustic Wave (SAW) sensors. When volatile organic compounds (VOCs) adsorb onto the coated surface, the resultant mass change leads to a measurable shift in the crystal’s resonant frequency. Owing to their viscoelastic characteristics, ionic liquids have emerged as a promising coating material for Quartz Crystal Microbalances (QCMs) in recent scientific investigations [110,111]. Zhang et al. designed a QCM sensor based on a chitosan-halloysite nanotube composite that improved the sensor’s selectivity and repeatability [112]. Figure 4 shows the preparation process of the CS-HNTs film-coated QCM humidity sensor.

These coating materials have also improved long-term stability and repeatability; however, more improvements need to be made to increase sensor lifespan. Additionally, certain coating materials, such as graphene-based products, may be difficult to fabricate, lack surface uniformity, or be expensive [113]. The straightforward design of QCM systems has facilitated the development of multiple commercial modules. However, their reliance on mass detection for VOC sensing can compromise selectivity due to susceptibility to nonspecific interactions. To address this, QCMs can employ various coating layers, including metal oxides, polymers, and other materials, to control VOC sensing capabilities [110,114,115].

QCM sensors are known for their long lifespan, low LOD, and ease of modification, making them versatile and portable. However, they require precise calibration, are sensitive to humidity and temperature, and have a limited measurement range and poor reproducibility, which can restrict their utility.

#### 2.2.2. Surface Acoustic Wave Sensors

**Figure 5:**
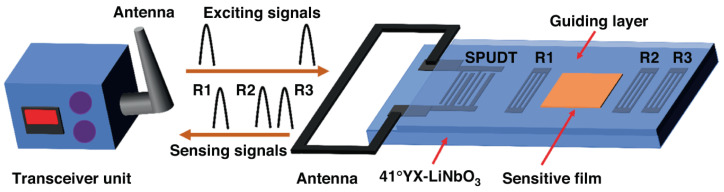
Schematic and working principles of the proposed SAW chemical sensor [121]. Reprinted from [121], Copyright (2024), with permission from Springer Nature Open Access Creative Common CC BY license.

Resonators have emerged as the leading choice for gravimetric VOC sensing due to their simplicity of fabrication. Surface Acoustic Wave (SAW) devices operate on the principle of a standing wave formed between interdigitated electrodes on a piezoelectric material’s surface. Exposure to VOCs alters the frequencies of waves traversing the SAW device surface, inducing changes in conductivity, stress, mass loading, and viscoelasticity. Various coating layers with different affinities, including metal oxides, graphene, polymers, and carbon nanotubes, have been employed to enhance the performance of SAW VOC sensors [116]. Gao et al. introduced a novel SAW variant termed dual transduction SAW, which detects variations in both mass and resistance within the sensing material. By leveraging the interplay between these factors, they successfully distinguished between different VOCs [117]. Viespe et al. demonstrated a significant enhancement in SAW sensor sensitivity by incorporating nanoparticles into the polymer sensing film [118]. Similarly, Kus et al. achieved notable sensitivity improvements by employing functionalized gold nanorods [119]. Palla-Papavlu et al. conducted a comprehensive review focusing on sensitive materials and coating technologies for SAW sensors [120]. Pan et al. developed a 433 MHz passive wireless surface acoustic wave (WSAW) gas sensor for detecting dimethyl methylphosphonate (DMMP) to address the challenge of long-term monitoring of environmental warfare agents with chemical gas sensors. The sensor features a YZ lithium niobate (LiNbO_3_) substrate with metallic interdigital transducers (IDTs) and a DMMP-sensitive viscoelastic polymer fluoroalcoholpolysiloxane (SXFA) film, operating effectively between −30 °C and 100 °C with humidity less than 60% RH. It achieved a lower detection limit of 0.48 mg/m^3^ and a sensitivity of 4.63°/(mg/m^3^). Notably, the sensor operates without batteries, can be used in hazardous areas, and is lightweight, small, and capable of withstanding harsh environmental conditions. The schematic and working principle of the sensor are illustrated in Figure 5 [121].

SAW sensors are versatile, portable, and cost-effective, with fast response times and long lifespans. Like QCM sensors, they are sensitive to temperature, have limited measurement ranges, and suffer from noise and poor reproducibility, which limits their precision and reliability.

#### 2.2.3. Thermal Sensor (Pellistors)

**Figure 6:**
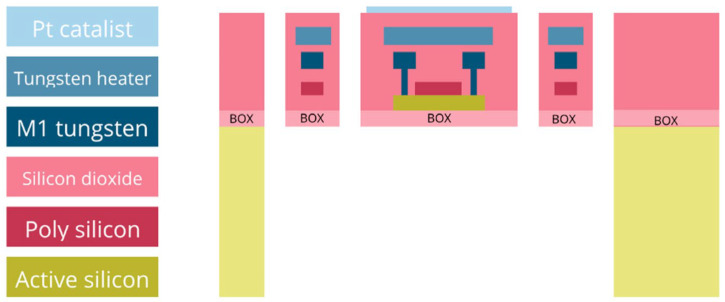
Schematic of a GMOS sensor [127]. Reprinted from [127], Copyright (2023), with permission from MDPI Open Access Creative Common CC BY license.

Pellistors are solid-state devices designed to detect combustible gases or gases that exhibit a significant difference in thermal conductivity compared to air. These sensors feature small catalyst-loaded ceramic “pellets” whose resistance changes in the presence of target gases, combining the elements of a “pellet” and a “resistor” to form the term “pellistor”. Typically, the detection limit for calorimetric sensors like pellistors is in the ppm range. Pellistors are categorized into two types: catalytic and thermal conductivity (TC). Catalytic pellistors function by measuring the heat generated from the catalytic oxidation of gas analytes, making them more commonly used in commercial settings. They detect gases by combusting the target gas, thus measuring specific combustion enthalpies and allowing for the detection of low concentrations in short response times. However, catalytic pellistors are sensitive to changes in the environment. On the other hand, TC sensors operate based on the thermal conductivity of gases, using either a platinum resistance temperature detector or a thermistor to measure temperature changes induced by gas interactions. Originally developed as successors to the flame safety lamp for detecting combustible gases, catalytic sensors offer enhanced accuracy and integration into complex detection systems. However, they are prone to catalyst poisoning caused by specific impurities in gas samples, which can lead to a severe and sometimes irreversible reduction in catalyst activity. Consistent maintenance is required to ensure safe handling of these sensors [122,123,124]. To address pellistors’ inadequate sensitivity and responsivity due to their passive nature, Nemirovsky et al. developed a novel type of thermal sensor called the thermal transistor MOS (TMOS), using the standard CMOS-SOI process and released through post-etching [125]. The TMOS offers high responsivity due to its built-in transistor amplification and subthreshold operation, making it suitable for a wide range of battery-powered applications. Their paper introduces a new gas sensor, named GMOS, which is based on TMOS technology. GMOS functions as a catalytic gas sensor, like pellistors, detecting combustible gases in the air. The use of CMOS-SOI technology and tungsten metallization allows GMOS to operate at very high temperatures (up to 450 °C in testing). Both the sensors and the readout circuitry are fabricated using the same CMOS-SOI process. In another study, they focused on understanding the sensor’s performance under high humidity conditions, using advanced computational fluid dynamics (CFD) simulations to model the effects of humidity on operating temperature and sensing response. The cross-section scheme of the GMOS sensing pixel deposited above the buried oxide (BOX) is shown in Figure 6 [125,126,127].

Pellistors are small, cost-effective, and durable, with fast response times, making them suitable for harsh conditions. However, their moderate limit of detection and sensitivity to environmental changes necessitates maintenance and calibration, reducing their versatility in complex environments.

#### 2.2.4. Optical Methods

Optical chemical sensors are sophisticated devices used to detect and analyze chemical interactions through the modulation of radiation intensity across infrared, visible, or ultraviolet spectra. These sensors typically consist of a light source, a substrate or sample cell where analytes interact with the light, and a detector that captures changes in specific wavelengths. This technology leverages different optical properties such as absorption, scattering, and luminescence to identify various chemical substances. In this discussion, we will explore examples of optical sensors, including nondispersive infrared sensors (NDIRs), chemiluminescence sensors, colorimetric sensors, and fluorescent VOC sensors, to illustrate the range and utility of these technologies [128].

Incorporating optical sensors in the detection of volatile organic compounds (VOCs), fluorescent VOC sensors exemplify precision through their ability to detect subtle changes in fluorescence emissions. These sensors, which are critical in monitoring low concentrations of chemical entities, evaluate parameters such as emission peak shifts, intensity changes, and fluorescence lifetimes. This capability provides a robust analytical platform suitable for detecting a broad spectrum of VOCs, demonstrating high sensitivity and specificity essential for various applications [129,130]. The main challenge in fluorescent sensor development is their limited anti-interference ability. To improve sensitivity and reduce the effect of background interference, carbon dots have proven to be promising [131]. Yang et al. utilized a red emissive carbon dot-based fluorescent sensor to monitor polarity changes in biological systems with minimal interference from background fluorescence [132]. Keerthana et al. synthesized dual emissive carbon dots to correct environmental interference and improve selectivity [133]. In a recent study, Liu et al. developed a Ni(II)-MOF-based luminescent sensor for the detection of the 3-nitrotyrosine (3-NT) biomarker and 6-propyl-2-thiouracil (6-PTU) anti-thyroid drug in urine. The sensor exhibited strong fluorescence at 448 nm upon excitation at 336 nm, showing excellent sensitivity and selectivity for the target analytes. Notably, the sensor was unaffected by common urine components, highlighting its potential for non-invasive, real-time diagnostics in disease monitoring and early detection [134]. On a simpler but equally effective front, colorimetric sensors employ a straightforward optical response to chemical or physical stimuli. This direct visual cue, augmented by the use of ubiquitous digital color detection technologies, renders these sensors particularly helpful for immediate on-site analyses and real-time monitoring. Commonly applied across environmental monitoring, healthcare diagnostics, and food safety, colorimetric sensors are valued for their simplicity and rapid response [129,135,136,137,138]. Chemiluminescence (CL) sensors are another innovative approach within optical sensing technologies. These sensors operate based on the principle of light emission during chemical reactions, such as those between ozone and various nitrogen or sulfur-containing compounds. The unique ability of CL sensors to offer exceptionally low detection limits, alongside their capability to distinguish between different compounds via specific light emission wavelengths, positions them as highly effective tools for identifying and tracing environmental pollutants. Integration with analytical methodologies like gas chromatography further enhances their specificity and sensitivity, making them invaluable in sophisticated detection systems [139,140].

Ohira et al. utilized CL to measure isoprene levels in human breath, while Mukosera et al. detected dinitrosyl iron complexes, essential intermediates in NO metabolism, using ozone chemiluminescence [141]. Zhao et al. introduced a CL method for determining chemical oxygen demand in water, noted for its environmental friendliness and rapidity [142]. Similarly, Matsumoto measured total ozone reactivity from BVOCs in forest air using CL [143]. Conversely, the chemiluminescence reaction of ozone can also gauge ozone concentrations, such as in the atmosphere when using isoprene gas as a reaction partner.

Additionally, nondispersive infrared sensors (NDIRs) provide targeted detection of gas-phase analytes through the absorption of infrared light, capitalizing on molecular vibrations and rotations. Unlike conventional infrared spectrometers, NDIR sensors utilize optical filters instead of diffraction gratings or prisms, allowing for selective analysis of spectral regions emitted from a broadband source. While NDIR sensors efficiently detect a variety of common gases, including CO_2_, CO, NO, NO_2_, SO_2_, H_2_S, and CH_4_, their semi-selective nature may lead to challenges in distinguishing overlapping absorption spectra, necessitating careful calibration and setup. This careful setup often requires expensive and large instrumentation, making NDIR sensors difficult to miniaturize. Each of these sensor types leverages unique optical properties to fulfill critical roles in diverse applications, highlighting the expansive utility and adaptability of optical chemical sensors in modern analytical and diagnostic practices [144,145].

**Figure 7:**
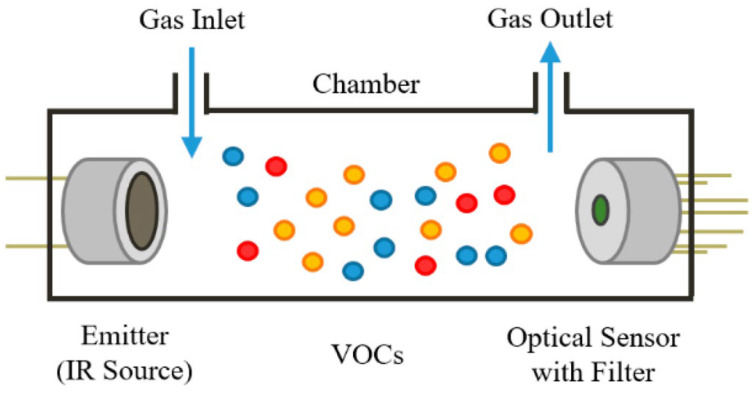
Optical gas sensing principle [148]. Reprinted from [148], Copyright (2020), with permission from MDPI Open Access Creative Common CC BY license.

Tan et al. introduced a multiplexed NDIR gas sensing platform featuring a narrowband infrared detector array, enabling multi-gas sensing without the need for bulky bandpass filters and detectors [146]. Similarly, Xu et al. demonstrated a similar system capable of simultaneous analysis of multiple automobile exhaust gases [147]. Esfahani et al. showcased the versatility of NDIRs as sensors in electronic noses, addressing issues such as sensor drift, poor repeatability, lack of robustness, replicability, and susceptibility to temperature and humidity effects. A nondispersive infrared (NDIR) sensor system consists of three main parts: the emitter (IR source), gas flow path, and IR detector can be seen in Figure 7 [148].

All in all, NDIR sensors offer high stability, low power consumption, and real-time response, making them suitable for long-term use in portable applications. However, they are limited by their inability to miniaturize easily and their sensitivity to environmental factors, making them more specialized for specific gas detection.

Another optical sensor with promising results is the Raman sensor. These sensors utilize Raman spectroscopy, a technique that involves using a laser to excite the molecules in a sample. When the laser interacts with these molecules, the majority of the light scatters elastically, but a small portion scatters inelastically, causing a shift in wavelength. The Raman sensor detects these shifts to generate a spectrum, with each peak providing a ‘fingerprint’ of the VOCs present. These sensors have been shown to be highly sensitive, but they suffer from difficult instrumentation and often require complex algorithms to process data [149,150]. The specificity of these sensors is partly due to the development of surface-enhanced platforms. Zhao et al. successfully developed a surface-enhanced Raman sensor (SERS) for the detection of formaldehyde by incorporating gold nanorods, achieving a limit of detection as low as 0.86 nM [151]. Xu et al. developed a flat graphene surface with a plasmonic metal nano-island array that provided increased strong magnetic hotspots for Raman enhancement and greater stability [152]. Ding et al. developed a novel film that can be applied to SERS for rapid, in situ detection of carbaryl, providing a low limit of detection and affordable fabrication [153]. Other methods of sensor enhancement have been explored. Velez et al. utilized a multimode blue laser diode to enhance trace gas sensing [154], while Hanf et al. explored utilizing fiber-enhanced Raman spectroscopic for a versatile gas sensing and breath analyzer [155]. While challenges remain in fully optimizing these systems, their potential selectivity and specificity make these sensors a compelling choice.

#### 2.2.5. Photoionization Detectors

**Figure 8:**
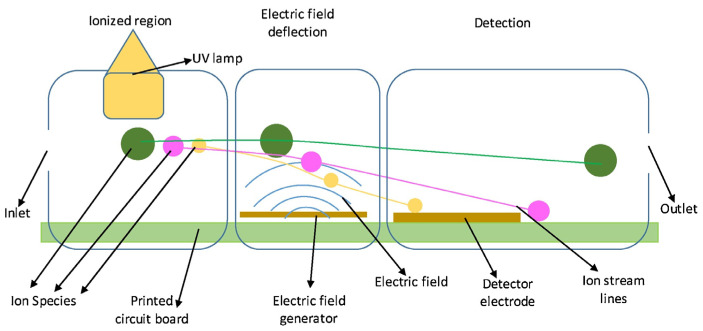
System illustration depicting the ionization, electric field, and detection regions within the sensor [156]. Reprinted from [156], Copyright (2018), with permission from Elsevier.

Photoionization detectors (PIDs) utilize UV light to ionize gas molecules, effectively ionizing most organic compounds without affecting the primary components of air. They can detect a wide range of volatile organic compounds (VOCs) and some inorganic gases like ammonia and hydrogen sulfide, but they are ineffective with methane and other very low molecular weight VOCs. The ions generated produce an electric current at an electrode, which serves as the detector output. PIDs have long been used to monitor worker exposure to VOCs across various industries, and research into enhancing their capabilities continues. PID sensors provide a concentration value of the sample being tested, without any additional information of the chemical composition. However, Covington and Agbroke have developed a PID sensor that can differentiate between some VOCs and provide compositional insights. The sensor features detection electrodes on one side and signal processing electronics on the other, with an ionization chamber and fluidics fitted over the detectors [156]. A system illustration can be seen in Figure 8.

Similarly, Pang et al. have investigated replacing flame ionization detectors (FID) with PIDs in gas chromatography (GC), finding them comparably effective with the added benefits of better portability and no reliance on hydrogen. They also explored using different lamps to enhance functionality [157].

Additionally, Spadi et al. conducted studies with a standalone PID, successfully classifying different rosemary species by analyzing the temporal kinetics of their VOC emissions, which provided unique ‘fingerprints’ for each variety [158]. Like PIDs, flame ionization detectors (FIDs) are used for VOC detection, typically as GC detectors or standalone instruments. While FIDs are more responsive to carbon chains, PIDs excel in detecting functional groups, with the detection signal being proportional to the number of non-oxidized carbon atoms. Despite the continued use of FIDs in total VOC analysis, innovation in this area has shifted, largely due to the limitations of requiring hydrogen gas for operation [87,159]. PIDs provide real-time responses, low limits of detection, and portability, but their sensitivity to humidity, inconsistent performance, and limited gas detection range hinder their overall performance in complex environments. Additionally, their poor selectivity limits their ability to distinguish between similar gases, further affecting their effectiveness in diverse or mixed gas environments.

#### 2.2.6. Gas Chromatography Coupled with Mass Spectrometer and Ion Mobility Mass Spectrometer

**Figure 9:**
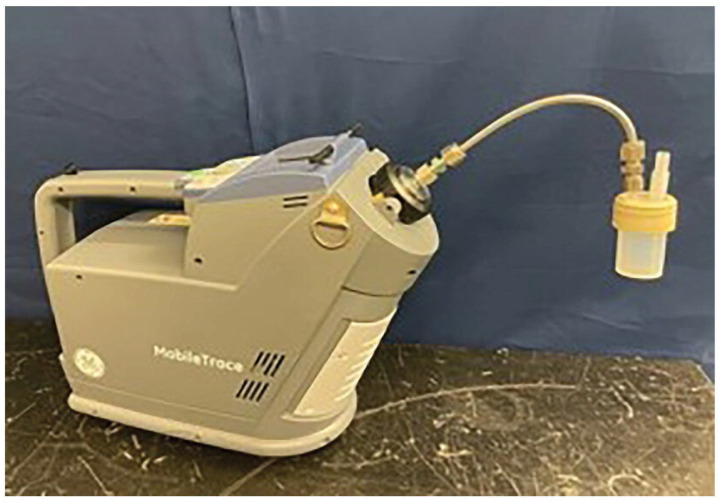
Non-contact detection of fentanyl by a field-portable ion mobility spectrometer [169]. Reprinted from [169], Copyright (2018), with permission Wiley and Sons.

Gas chromatography coupled with mass spectrometry (MS) is highly valued in analytical chemistry for its accuracy in identifying molecular weights and structures, with high-resolution versions capable of pinpointing exact molecular formulas. Although traditional MS systems are generally large and power-intensive, they consist of three key components: an ion source, a mass analyzer, and a detector [160]. Recent advancements have greatly improved ion sources, yet the portability of these systems is still limited by the mass analyzer, which needs vacuum conditions and significant space for ion separation. Moreover, simplifying the operational complexity is crucial for effective use in the field. Despite these challenges, there have been advancements in developing portable MS systems, especially for detecting volatile organic compounds (VOCs). This paper discusses the progress in standalone MS and Ion Mobility Spectrometry (IMS) technologies, emphasizing their potential for portable applications [161,162]. Gas chromatography–mass spectrometry (GC-MS)-based sensors have gained increasing interest over the last few years due to their accuracy and fast performance. Allsworth successfully developed a GC-MS-based sensor with sub 1 ppm mass accuracy across various VOCs, including benzene, toluene, ethylbenzene, and o-xylene [163]. Vietro et al. developed a GC-MS-based system that could potentially diagnose transplanted kidney rejection through VOC monitoring by detecting increases in concentration [164]. Ion Mobility Spectrometry (IMS) combines elements of gas chromatography (GC) and mass spectrometry. It differs from traditional MS by performing ion separation at atmospheric pressure instead of in a vacuum. In this technique, ions move through a gas medium, typically air, and their separation is based on their mobility, which is influenced by their mass-to-charge ratio and shape. IMS is very adaptable; when paired with ionization technologies such as radioactive H electron emitters, X-ray ionization units, or atmospheric pressure chemical ionization (APCI), it becomes compact enough for handheld devices. This flexibility allows IMS to function either as a standalone tool or together with GC, improving ionization processes and adding more analytical dimensions [165,166,167]. A significant development in IMS technology is the simplified, miniaturized drift tube designed by Ahrens et al., which has been incorporated into a standalone, battery-powered portable device [168]. In practice, Fulton et al. demonstrated that a handheld IMS device can detect the drug fentanyl by measuring its vapor form of N-phenylpropanamide, avoiding direct contact with the substance [169]. The overall schematic of the handheld IMS can be seen in Figure 9.

Additionally, Ratiu et al. used an aspiration IMS to distinguish between metabolic VOCs emitted by bacteria, applying statistical methods to analyze the IMS fingerprints [170]. Furthermore, research by Guo et al. comparing GC-MS and GC-IMS in analyzing oolong teas revealed 27 unique VOCs identified solely through IMS, showcasing its potential when combined with other mass spectrometry techniques [171].

Gas chromatography coupled with mass spectrometry and Ion Mobility Spectrometry provide highly sensitive and selective detection with low limits of detection, but they are expensive, energy-consuming, and require regular calibration and maintenance, making them impractical for many portable or low-cost applications.

## 3. Functionalized MIP-Based Sensors

Molecularly imprinted polymers (MIPs) consist of synthetic polymers crafted from functional monomers and cross-linkers. These sensors can be tailored with specific sites and structures to accommodate diverse classes of target analytes, with cavities within MIP networks customized to capture targets within specific sizes, shapes, or functional group ranges [172,173]. Their versatility has been demonstrated in various sensing fields, spanning chemical and biosensing applications [174]. However, despite their immense potential in chemical sensing, their application in detecting volatile organic compounds (VOCs) has been insufficiently explored [175]. The evolution of molecularly imprinted polymers (MIPs) has encompassed various methodologies, including bulk, precipitation, emulsion polymerization, sol-gel techniques, and electro-polymerization. In terms of combining MIPs with transducers, these approaches can be categorized into ex situ and in situ methodologies. Ex situ methods involve immobilizing pre-made MIP particles onto a transducer through physical treatments like drop-casting or spin-coating, or via chemical coupling [176].

**Figure 10:**
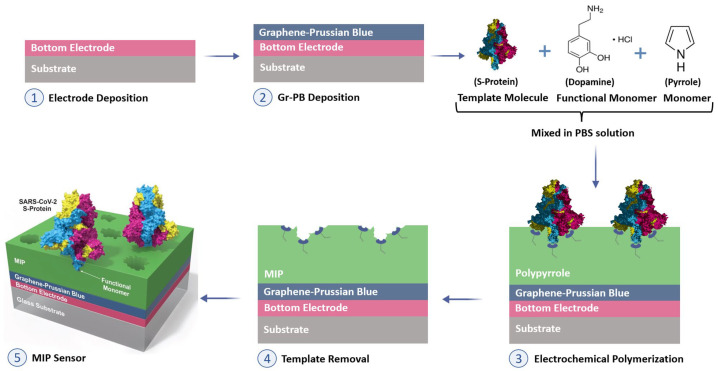
Schematic of the fabrication process and testing of the pathogen sensor, which employs functional monomers and template molecules (SARS-CoV-2 spike proteins) to create an artificial antibody (lock) designed specifically to detect SARS-CoV-2 pathogens (key) with high specificity [71]. Reprinted from [71], Copyright (2023), with permission from Elsevier.

Since 2017, our team has been developing functionalized molecularly imprinted polymers (MIP)-based sensors for biomarker VOCs aimed at diagnosing various diseases. Related studies by Emam et al. have focused on conditions such as Alzheimer’s disease [93] and lung cancer [177]. As soon as COVID-19 was declared a pandemic in early 2020, our team initiated the development of functionalized molecularly imprinted polymer (MIP)-based electrochemical sensors. These sensors are designed to detect SARS-CoV-2 pathogens in aerosols, forming the basis for a COVID-19 breathalyzer [71]. Our initial approach involved using the full-length spike proteins of SARS-CoV-2 as template molecules to develop these MIP sensors. This strategy achieved high sensitivity but resulted in relatively poor specificity. Since full-length spike proteins have molecular weights in the range of 180–200 kDa [178], which are too large for MIP sensors, we tried to use the S1 subunit of the spike proteins of the SARS-CoV-2 with molecular weights of nearly 78 kDa [179]. At the same time, we used functionalized MIP sensors by using 1-pyrenebutyric acid N-hydroxy succinimide ester (PBSE) and cysteamine to bind the S1 proteins before using the functionalized S1 proteins as template molecules. This effort led to pathogen sensors for detecting SARS-CoV-2 from the aerosol with significantly improved sensitivity and specificity. To further improve the sensitivity and specificity, we used the RBD subunit of the spike proteins of the SARS-CoV-2, which further reduced the molecular weight to nearly 26 kDa, and successfully achieved ultra-high sensitivity by a new functional monomer of dopamine. With these background efforts in mind, we present, for the first time, the development of highly accurate electrochemical sensors for ultrafast detection of SARS-CoV-2 in aerosols, alongside innovative testing methodologies and algorithms. The sensors we developed target the omicron-variant receptor-binding domain (RBD) spike proteins, demonstrating over 99% accuracy across various SARS-CoV-2 variants. These sensors feature rapid response and brief recovery times, delivering results in under 30 s. Our approach utilizes a molecularly imprinted polymer that is specifically functionalized to enhance accuracy, operating on the principle that the ohmic resistance of the sensor changes in response to the presence of COVID-19 pathogens in aerosol samples [71]. Our fabrication process is shown in Figure 10.

Table 3 shows a comparison to other market-available methods (see Table 1). Our pathogen aerosol-based sensor proves to be a viable and effective option for rapid COVID-19 testing [71].

Table 4 compares the performance of wavelet-based and deep-learning-based models with human visual inspection and curve-fitting results. The deep learning model offers a better balance of true positive and true negative rates but is more complex. In low-computation scenarios, the wavelet-based classifier is a good alternative [71].

MIPs offer the ability to target specific molecules with high sensitivity, selectivity, and a low limit of detection, while also being portable and cost-effective. However, their performance can be affected by temperature and humidity, requiring careful management during fabrication and use.

## 4. Discussion and Outlook

Many of the methods discussed earlier are susceptible to variations in humidity, temperature, or atmospheric pressure, which can significantly alter the performance and reliability of the detection method. Addressing these environmental factors is critical for ensuring accurate and consistent outcomes. Various solutions, such as incorporating humidity control systems, have been explored to mitigate these issues. Researchers like Paknahad et al. have developed humidity removal membranes for microfluidic-based gas-sensing devices to mitigate environmental influences [180]. Additionally, temperature and pressure regulating systems could be incorporated into sensor setups to further enhance stability. Sensor arrays with individual selectivity have been commonly employed in these methods. Nevertheless, these systems introduce complexities in analysis, demand frequent calibration, and may require component replacements due to sensor drifts. As such, there is still a demand for simpler, more reliable sensors [181].

The capabilities of the analytical methods mentioned in this article are summarized in Table 5, which summarizes the suitable analytes for each method along with their respective advantages and limitations. For the detection of volatile organic compounds (VOCs), electrochemical sensors and nondispersive infrared sensors (NDIRs) are superior choices. Electrochemical sensors offer a broad detection range, low power consumption, durability, and a rapid response time, making them highly versatile for various applications. These sensors are cost-effective and operate efficiently at room temperature, with good limits of detection (LOD) for low concentrations of VOCs. Despite their limitations, such as a limited shelf life and baseline instability, their advantages make them suitable for real-time monitoring. Continuously, NDIR sensors provide fast response times, long-term stability, and insensitivity to environmental changes, which are crucial for consistent and reliable VOC detection. Although these sensors tend to be more expensive and challenging to miniaturize, their high selectivity and stability make them ideal for applications requiring precise and long-term monitoring. Together, these technologies offer a balance of sensitivity, reliability, and operational efficiency, making them the leading choices for modern VOC detection needs.

It is important to note that therapeutic agents and biosensors designed for VOC biomarker detection face challenges related to off-target effects, where non-target compounds interfere with detection, and poor selectivity. To address these issues, one viable solution is the use of highly selective sensors based on molecularly imprinted polymers (MIPs), which can be tailored to bind only to specific biomarkers, reducing interference from other VOCs. Additionally, advanced computational techniques like in silico modeling can help predict potential cross-reactivity with other compounds before sensor development, ensuring higher specificity [207]. Moreover, multi-sensor arrays can be employed to cross-check readings from different sensors, helping to mitigate off-target effects by providing more comprehensive data on the sample. Lastly, developing biodegradable and biocompatible sensor materials, such as biodegradable polymers like polylactic-co-glycolic acid (PLGA) or natural fiber composites, ensures that the sensors are safe, environmentally friendly, and minimally invasive, while maintaining their performance in various clinical environments [208]. This approach also reduces concerns about poor biodegradability, enhancing the sustainability of the sensors.

## 5. Conclusions

This review provides a thorough examination of VOC sensing, emphasizing the latest progress in the field. It explores state-of-the-art innovations in sensing materials along with the advent of new sensing systems and applications. The review highlights promising advancements while addressing the significant challenges that remain. Although there has been notable progress, VOC sensing technologies still need to fulfill the requirements of varied applications, such as detecting diseases, wearable monitoring devices, and detecting indoor toxic VOCs. Future VOC sensors would focus on improving sensitivity and selectivity through advanced materials, innovative structures, and the application of machine learning techniques. With the spread of affordable and miniaturized sensing components, VOC sensors are poised to become widespread in various settings. Looking forward, the development of superior sensing materials and novel applications is anticipated. Achieving these goals will require extensive research to overcome the hurdles that limit the commercial potential of emerging VOC sensing technologies.

## Figures and Tables

**Table 3:** A comparison between the novel sensors developed in this work with those already available on the market for COVID-19 testing [71].

Method	Approach	Sensitivity	Specificity	Time	Body Fluids	Vendors
RT-PCR	RNA	95–100%	95–100%	3 h	Respiratory Specimens	LabCrop, Roche, etc.
Antibody	IgM, IgG	80–100%	90–100%	15–30 min	Blood	Cellex, etc.
Antigen	RNA	20–71%	85–100%	15–30 min	Blood	UCSD
CRISP-based	RNA	95%	100%	40 min	Respiratory specimens	Sherlock Biosciences
LAMP-based	RNA	97%	100%	30 min	Respiratory specimens	MicrosensDx
Saliva-based	RNA	91%	98%	45 min	Saliva	Yale School of Public Health
Breath-based	GC-MS VOC	91%	99%	5–10 min	Air	Inspect-IR, Breathomix
Our Sensor	RBD S-Protein	98.40%	100%	10 s	Aerosol/Air	Winchester Technologies
Antibody	IgM, IgG	80–100%	90–100%	15–30 min	Blood	Cellex, etc.
Antigen	RNA	20–71%	85–100%	15–30 min	Blood	UCSD
CRISP-based	RNA	95%	100%	40 min	Respiratory specimens	Sherlock Biosciences

**Table 4:** A comparison between the human-brain decision with the outputs of three different theoretical classifying methods in terms of sensitivity, specificity, and accuracy (obtained based on the sensor’s R(t) profiles) for a sensor exposed to a positive test kit [71].

Method	Sensitivity	Specificity	Accuracy
Human-Brain Decision	98.41%	100%	99.26%
Wavelet Method	92.10%	90.30%	91.10%
Deep-Learning Method	95.20%	90.30%	92.60%
Curve-Fitting Method	95.23%	100%	97.78%

**Table 5:** An overview of analytical methods, focusing on their sensitivity, selectivity, and the associated advantages and limitations.

Sensor Type	Sensitivity	Selectivity	Comments	Reference
Photoionization Detectors (PID)	++++	++	+ real-time response + low LOD + cost-effective + portable and simple to use –- requires maintenance – sensitive to humidity – limited gas detection – inconsistent response	[109,182,183,184]
Electrochemical Sensors	+++++	++++	+ broadband sensors + low power consumption + durable + real-time response + room temperature operation + cost-effective + low LOD + rapid response – limited shelf life – cross-sensitivity to other gases – requires maintenance – low baseline stability	[184,185,186,187]
Chemiresistors	+++	++	+ good durability + portable + cost-effective + simple and easy to use + real-time response + non-destructive + portable + responds to significant number of gases – influenced by humidity and temperature – high working temperature – not very flexible – more fragile, easily damaged during preparation	[184,188,189,190,191]
Quartz Crystal Microbalance (QCM)	++++	+++	+ real-time response + cost-effective + long lifetime + non-destructive + low power consumption + ease of modification + portable + low LOD – humidity/temperature sensitive – limited measurement range – requires precise calibration – limited to specific target molecules – poor reproducibility	[184,192,193]
Surface Acoustic Wave (SAW)	++++	+++	+ versatile + portable +cost-effective + low power consumption + high-frequency operation + fast response + long lifetime – limited measurement range – temperature sensitive – signal-to-noise ratio – noisy – poor reproducibility – limited to specific target molecules	[167,177]
Nondispersive infrared sensors (NDIRs)	+++	+++++	+ real-time response + long-term stability + portable + low power consumption – difficult to miniaturize – limited to specific gases – humidity/temperature sensitive – expensive instrumentation	[136,137,167]
Raman Sensors	+++	++++	+ non-destructive + minimal sample preparation + high specificity + real-time response – expensive instrumentation – limited portability – complicated data analysis	[149,150,151,152]
Chemiluminescence Sensors	++++	+++	+ lower background emission than fluorescent sensors, avoiding noise caused by light scattering. + low LOD + detects chemicals with short lifespan + versatile with many catalysts + stable in most conditions + easy to use + has cost-effective options – irreversible exposure limits lifespan – reagents are expensive and often poisonous – conventional sensors only operate within 400–850 nm range – limited commercial availability – often limited portability	[194,195,196,197,198]
Fluorescent Sensors	++++	+++	+ rapid response + portable + real-time monitoring + simple operation + strong reversibility and recovery post-exposure + stable, withstands range of temperatures and pH variations – limited portability – more background interference compared to other sensors	[199,200,201,202]
Gas Chromatography Coupled with Mass Spectrometer and Ion Mobility Mass Spectrometer	+++++	+++++	+ fast response + low LOD – expensive – high energy consumption – operates well in ambient conditions – Regular calibration and maintenance needed – tends to struggle with certain chemicals like alkenes – large instruments	[101,203]
Molecularly Imprinted Polymers (MIPs)	+++++	+++++	+can be designed to operate in various environments. + cost-effective + portable + low LOD – limited to the specific molecules for which they are imprinted – sensitive to temperature and humidity – requires careful template removal	[66,156]
Pellistors	+++	++++	+ small + cost-effective + fast response time + cost-effective + durable in harsh conditions – moderate LOD – sensitive to environment changes – maintenance required	[102,204]
Colorimetric	+++	++++	+ disposable + low LOD + simple to use + portable + cost-effective + easy fabrication + operate at room temperature – susceptible to environmental changes – slower response time compared to some advanced sensor types – may require frequent recalibration depending on the application	[193,205,206]

Note: “+” indicates advantages, and ““ indicates disadvantages in this table. The selectivity and sensitivity are also represented by the number of “+” signs.

## Data Availability

The raw data supporting the conclusions of this article will be made available by the authors on request.
